# Using an ultraviolet cabinet improves compliance with the World Health Organization’s hand hygiene recommendations by undergraduate medical students: a randomized controlled trial

**DOI:** 10.1186/s13756-020-00808-4

**Published:** 2020-09-03

**Authors:** Sandrine Dray, Samuel Lehingue, Sabine Valera, Philippe Nouguier, Michel Salah Boussen, Florence Daviet, Delphine Bastian, Estelle Pilarczik, Isabelle Jousset, Sébastien Le Floch, Georgette Grech, Georges Leonetti, Laurent Papazian, Nadim Cassir, Jean-Marie Forel

**Affiliations:** 1 Médecine Intensive Réanimation, Assistance Publique Hôpitaux de Marseille, CHU Nord, Chemin des Bourrely, 13015 Marseille, France; 2 Service d’Aide Médicale Urgente (SAMU), Assistance Publique Hôpitaux de Marseille, CHU Timone, Marseille, France; 3 Département d’Anesthésie Réanimation, Assistance Publique Hôpitaux de Marseille, CHU Timone, Marseille, France; 4 Service d’Accueil des Urgences, Assistance Publique Hôpitaux de Marseille, CHU Nord, Marseille, France; 5 Comité de Lutte Contre les Infections Nosocomiales (CLIN), Assistance Publique Hôpitaux de Marseille, CHU Nord, Marseille, France; 6 Faculté de Médecine de Marseille, Aix Marseille University, Marseille, France; 7 Faculté de Médecine de Marseille, EA 3279, CEReSS - Health Service Research and Quality of life Center, Aix Marseille University, Marseille, France

**Keywords:** Hand hygiene, Education, Infection prevention, Alcohol-based handrubs, Ultraviolet-cabinets, Medical student, Health simulation

## Abstract

**Background:**

Appropriate hand hygiene (HH) is key to reducing healthcare-acquired infections. The World Health Organization (WHO) recommends education and training to improve HH knowledge and compliance. Physicians are ranked among the worst of all healthcare workers for compliant handrubbing with its origin probably being the failure to learn this essential behavior during undergraduate medical studies. This study evaluated if the use of Ultraviolet-cabinets (UVc) for fluorescent-alcohol-based handrubs (AHR) during an undergraduate medical student training improved the compliance rate to the WHO hand hygiene recommendations (completeness of AHR application and HH opportunities).

**Methods:**

This randomized trial compared a HH training with personal feedback (using UVc) to a control group. The first year, the students (2nd degree) were convened by groups (clusters) of 6–9 for a demonstration of the correct execution of WHO procedure. Randomization by cluster was done prior HH training. In the control group, the students hand rubbed under visual supervision of a tutor. In the intervention group after the same visual supervision, completeness of fluorescent-AHR hand application was recorded under UVc and was shown to the student. The intervention group had free access to the UVc until complete application. HH practices were included in simulation sessions for the both groups. One year after (3rd degree), all the students were asked to hand rub with fluorescent-AHR. A tutor (blinded to the study group) assessed the completeness of hand application under UVc and the compliance with the WHO opportunities. Complete application of AHR was defined as fluorescence for all the surfaces of hands and wrists.

**Results:**

242 students participated (140 in the intervention group and 102 in the control group). One year after the initial training, the rate of complete application of AHR was doubled in the intervention group (60.0% vs. 30.4%, *p* < 0.001). In a multivariate analysis which included gender, additional HH or UVc training, surgical traineeship and regular use of AHR, the hazard ratio for the intervention was 3.84 (95%CI: 2.09–7.06). The compliance with the HH WHO’s opportunities was increased in the intervention group (58.1% vs. 42.4%, *p* < 0.018).

**Conclusion:**

Using UVc for undergraduate medical students education to hand hygiene improves their technique and compliance with WHO recommendations.

## Background

Appropriate hand hygiene (HH) is key to both preventing pathogen transmissions and reducing healthcare-acquired infections. Recently, SARS Cov-2 transmission highlights the importance of hand hygiene. The World Health Organization (WHO) recommends education and training as part of a multimodal strategy to improve HH knowledge and handrubbing compliance. Physicians are ranked among the worst of all healthcare workers (HCW) for compliant handrubbing [1, 2] with its origin probably being the failure to learn this essential behavior during undergraduate medical studies. Despite the fact that past studies recommended an increased emphasis on HH in undergraduate teaching [1–5], very few studies to date explore the development and testing of HH education modules, particularly for the youngest medical students [6–8]. The focus is more on being compliant with HH indications than HH techniques. While compliant handrubbing for nosocomial infection prevention is more preponderant, poorly performed HH may also lead to pathogen transmission as outlined in the “how to handrub” section of the WHO guidelines [9]. These guidelines use an ultraviolet (UV) light inspection cabinet among the pedagogical tools to enhance alcohol-based handrubs (AHR). Supervised personal feedback states that the use of UV improves performance of the technique for short-term periods in medical and nursing students [10–13]. However, to the best of our knowledge, no randomized controlled trial (RCT) has evaluated such a strategy. Moreover, the long-term effects of these educational programs is poorly analyzed.

The aim of the present study was to evaluate, in a RCT, the long-term (one year) contribution of supervised personal feedback using UV light inspection cabinets in a HH education program for pre-internship second and third year undergraduate medical students.

Our main hypothesis was that the use of a supervised personal feedback for fluorescent AHR using a UV cabinet during the first year program would increase the rate of complete AHR handrubbing on the 2nd year. Our secondary hypothesis was that this could enhance compliant handrubbing according to WHO’s HH opportunities during simulation scenario-based learning activities in the second year of medical training.

## Methods

**Fig. 1:**
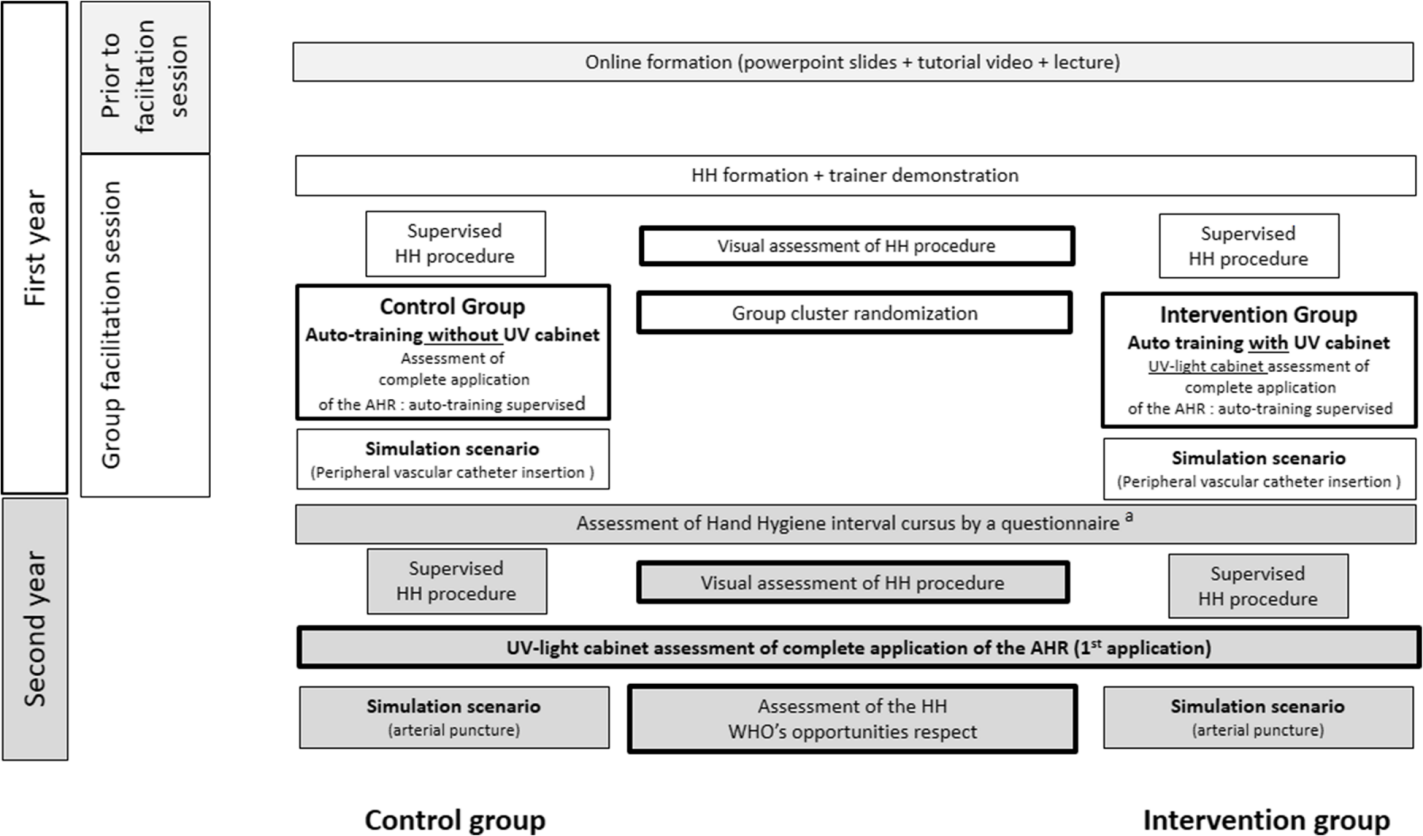
Study design. ^*a*^*:* New Hand hygiene training; Extra-university use of UV cabinet; infectious disease unit or surgical unit or intensive care unit traineeship; care of a patient needing contact precautions; care of a patient hospitalized for nosocomial infection; familiarity with alcohol-based solutions for hand hygiene. Abbreviations: HH: hand hygiene, UV: ultraviolet

This study was a cluster randomized trial in two parallel groups comparing training with supervised personal feedback using UV cabinet to a control group (without UV). The study was conducted between November 2015 and May 2017 during two years of a medical training program. This program was mandatory for second and third year undergraduate medical students prior to their internship at the Aix-Marseille University. The study was approved by our Institutional Review Board. An informed consent from each participant was required. Twenty students refused to participate. The study’s design is described in Fig. 1.

During the first year, and prior to their first simulation session, medical students had digital work station access to online PowerPoint slides as well as a short video extracted from the “WHO tools and resources” [14, 15]. The first module of the program occurred prior to their in-hospital traineeship. Simulation sessions by groups of six to nine students were set up to enhance learning HH practices. This was achieved via simulation scenario-based learning activities on common medical acts (insertion of a peripheral vascular catheter during the first year training and an arterial puncture during the second year training). The first module was partitioned into the four following steps: 1) a short lecture on the basics of HH, 2) an Infection Control Department tutor demonstrating the correct execution of WHO’s six-step HH procedures (Additional File 1), 3) HH training on AHR correct technique and finally, 4) a simulation scenario based on learning a peripheral vascular catheter insertion showing HH objectives and opportunities (“the five moments for HH in health care of WHO” i.e. before touching a patient, before clean/aseptic procedures, after body fluid exposure/risk, after touching a patient, and after touching patient surroundings) translated into practice. After the same HH review and demonstration the training differed between the two groups as follows:

- In the intervention group: Student used a fluorescent AHR and UV cabinet (Daro UV Systems©, England) and supervised personal feedback. Each student received the same visual assessment as the control group, and after being shown their levels of completeness for AHR application under the UV light, the tutor recorded those results as complete or incomplete fluorescence of hands. The student was then given open access to the fluorescent AHR and the UV cabinet to repeat the WHO’s handrubbing procedure, until both the tutor and the student judged their technique adequate.
- In the control group: Student handrubbed under the supervision of a tutor to assess their skills. The student was then given individualized recommendations and requested to handrub again until the tutor judged the completeness of AHR application according with the WHO’s handrubbing procedure had been achieved.

Each students group underwent a computer-generated cluster randomization into the control group or the intervention group of the study just before the HH training.

On the second year, the program was the same but without new reminder and demonstration.

The medical students completed a questionnaire assessing their HH training and other potential confounding factors during the time interval between the two simulation sessions (new HH formation, experiences related to HH during training, supplementary experiences with the UV cabinet) (Additional File 2). Before the beginning of the scenario-based learning for an arterial puncture, each student was asked to perform the WHO’s handrubbing procedure using the fluorescent AHR. The first year’s tutor was replaced by another who was blinded to the assigned group. On a standardized document, the tutor visually assessed and recorded the quality of the WHO’s handrubbing procedure; the completeness of hand hygiene by using UV light and finally their compliance with the WHO’s opportunities for HH during the simulation session (Additional Files 3 and 4). Main endpoint of the study was defined as the ratio between the number of students with a complete fluorescence of hands and the total number of students in the group. The percentage of complete fluorescence of hands was compared between the intervention group and the control group.

A senior hygienist physician (NC) supervised the educational program. Students’ training and evaluations were performed by four ICU physicians and four hygienist nurses, each with a HH post-graduate certification. These eight tutors were randomly assigned to a cluster. Preliminary meetings were conducted to standardize their evaluations of the WHO’s HH procedure, the completeness of handrubbing under UV light and the compliance with the WHO’s HH opportunities. The completeness of fluorescent AHR handrubbing was defined as fluorescence for all the surfaces of both hands and wrists. Compliance with HH opportunities was defined as the achievement of “the five moments for HH in health care” from the WHO during scenario-based learning (peripheral vascular catheter insertion and arterial puncture). A standardized document (Additional files 3 and 4) was used by tutors for the students’ evaluations. In order to calibrate the evaluation procedure, the eight tutors jointly evaluated the first twenty-one students. The degree of agreement between the tutors was carried out using the Fleiss kappa coefficient to test the interrater reliability.

### Statistical analysis

Quantitative variables were expressed as mean and standard deviations. Comparisons between control and intervention groups were performed using the Student t-test. Qualitative variables were expressed as the absolute value and percentage and compared by using the Chi^2^ Pearson test. Comparisons of the levels of completeness with AHR handrubbing and HH opportunities compliance between the first and the second-year data were tested using the Mc Nemar test. The associations between groups’ allocation and qualitative variables were assessed using the Chi^2^ Pearson test. During the second year, a multinomial logistic regression procedure was performed to identify factors associated with the completeness of AHR handrubbing and the compliance with the WHO’s HH opportunities using the control group as the reference. All of the variables with univariate test *p*-value < 0.20 were included in the logistic regression model. Statistical analysis was conducted using SPSS v.20.0 (IBM, New York, USA). Two-sided *p* value < 0.05 was significant.

## Results

### Characteristics of the students

**Fig. 2:**
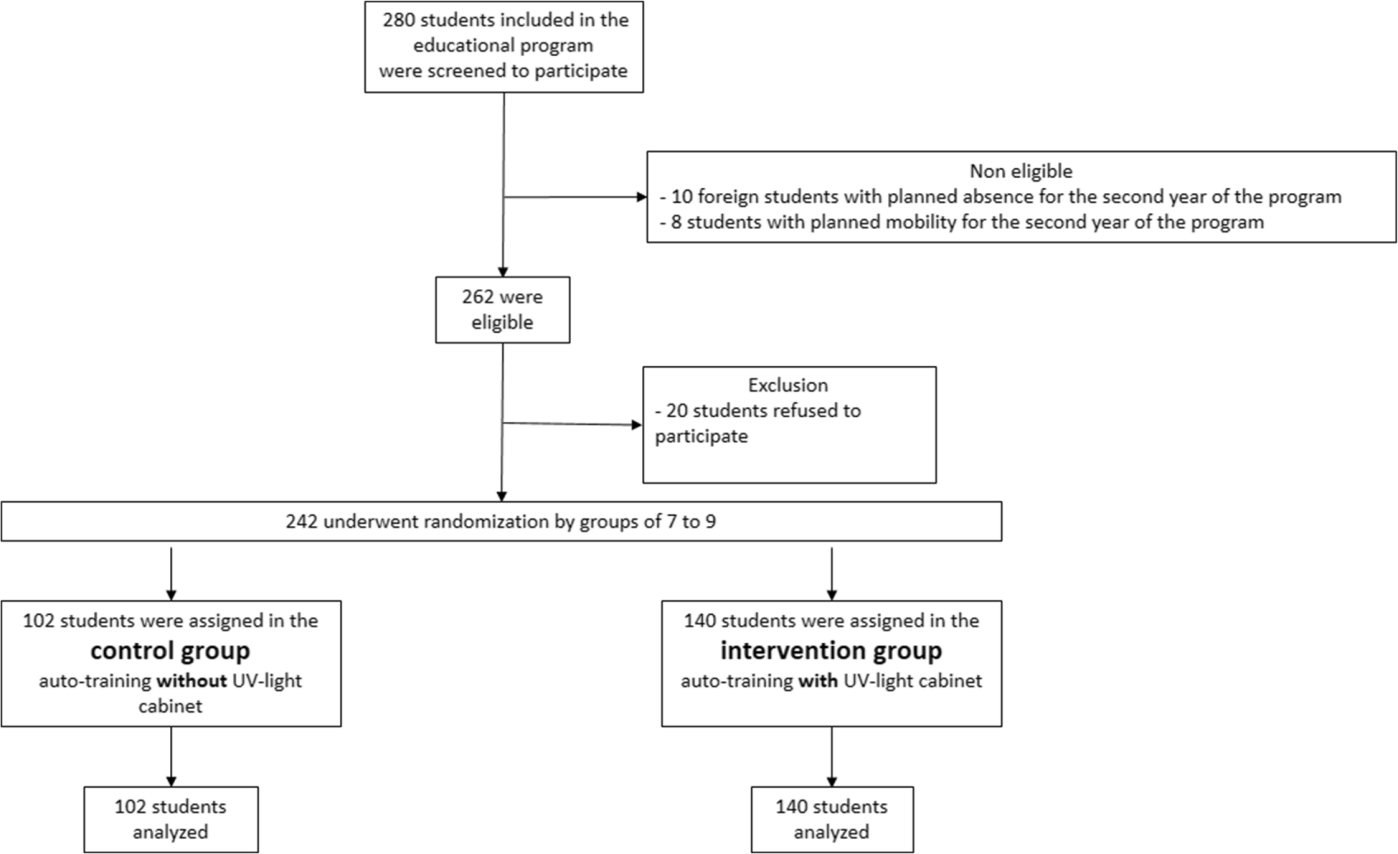
Study’s Flowchart. Enrollment, randomization and follow-up of the study participants

Among the 280 medical students eligible, 20 refused to participate, and 18 were ineligible because of missing data during their first or second year assessments. A total of 242 (86%) students were included in the analysis, with 140 being in the intervention group (17 student groups). The remaining 102 were in the control group (15 student groups) (Fig. 2). Baseline characteristics of students are presented in Table 1. The Fleiss Kappa coefficient showed an important agreement between the tutors (0.74 (95% CI: 0.66–0.82).

**Table 1:** Baseline characteristics of the students

Variable	All *n* = 242	Control group *n* = 102	Intervention group (Supervised personal feedback) *n* = 140	*p*-value
Age ± SD (years)	21.2 (±2.2)	21.0 (±1.8)	21.0 (±2.5)	0.36
Female gender	155 (64.0)	68 (66.7)	87 (62.1)	0.47
Hand hygiene personal experience before the study participation				
-Previous hand hygiene formation	13 (5.4)	5 (4.9)	8 (5.7)	0.78
-Previous use of UV cabinet	5 (2.1)	3 (2.9)	2 (1.4)	0.35
-Previous use of alcoholic solution for HH	57 (23.6)	25 (24.5)	32 (22.9)	0.65

Results are expressed as *n* (%)

Variables concerning the personal experience with hand hygiene before the study participation were collected on the first year as a questionnaire from each 2nd degree student (see Additional file 2)

### Main outcome: influence of supervised personal feedback on the completeness rate of AHR handrubbing

On the second year of pedagogical program, the 3rd degree students in the intervention group (supervised personal feedback with fluorescent AHR and UV cabinet) presented a higher rate of completeness for AHR application as compared with the 3rd degree students of control group (60.0% versus 30.4% *p* < 0.001, intervention vs. control groups).

Other factors significantly associated with the complete AHR application included supplemental training in a hospital surgical unit (*p* = 0.011) as well as the use of the UV cabinet for extra-university HH training (hospital training) in the interval between the first and second modules (*p* = 0.004) (Table 2). In the logistic regression model, the intervention group (those using the UV cabinet), women and the supplemental training in a hospital surgical unit were associated with an increased rate of complete application for the fluorescent-AHR (Table 2).

**Table 2:** Factors associated with the completeness of fluorescent alcohol-based handrubbing under ultraviolet-light (second year)

Variable	AHR Incomplete application (*n* = 127)	AHR Complete application (*n* = 115)	*p*-value	Hazard ratio	95% confidence interval	*p*’-value
	Univariate			Logistic regression		
Age ± SD (years)	21 ± 1.7	21 ± 2.7	0.68	–	–	–
Female gender	59.1	69.6	0.08	1.86	1.02–3.37	0.041
Personal feedback with UV cabinet	44.1	73.0	< 0.001	3.84	2.09–7.06	< 0.001
New Hand hygiene formation	31.5	43.8	0.057	1.59	0.82–3.09	0.17
Extra-university use of UV cabinet	14.5	30.4	0.004	1.18	0.54–2.58	0.67
Infectious disease unit traineeship	19.4	16.1	0.51	–	–	–
Surgical unit traineeship	54.5	70.5	0.011	1.85	1.00–3.43	0.049
Intensive care unit traineeship	10.5	13.4	0.49	–	–	–
Care of a patient needing contact precautions	70.2	75.0	0.41	–	–	–
Care of a patient hospitalized for nosocomial infection	16.1	15.3	0.86	–	–	–
Familiarity with alcoholic solution for HH	75.0	82.1	0.17	1.44	0.73–2.86	0.29

Results are expressed as %, except Age as mean (±SD)

*p*-value: comparison by univariate test between the group with AHR Incomplete application versus the group with AHR Complete application

*p’-value: p-value by the multivariate logistic regression*

*Completeness of AHR handrubbing:*

*-*AHR incomplete application corresponds to the presence of one or more areas without fluorescence on the hands and wrists of the students under UV-light

-AHR complete application corresponds to the absence of areas without fluorescence on the hands and wrists of the students under UV-light

Variables concerning the personal experience with hand hygiene between the first and the second year were collected on the second year as a questionnaire format from each 3rd degree student (see Additional file 2*)*

*Abbreviations: AHR: Alcohol-based handrub, SD: standard deviation; UV: Ultraviolet*

During the first year assessment (2nd degree students), only 43 of the 140 intervention group students (30.7%) had a complete AHR handrubbing by using the alcohol solution under UV-light before supervised personal feedback (first assessment). The use of supervised personal feedback with fluorescent AHR and UV cabinet by 2nd degree medical students, double their rate of complete AHR handrubbing one year later (60.0 vs. 30.7%, 2nd vs. 1st year). During the supervised personal feedback using UV cabinet and florescent AHR on the first year, the mean number of attempts before a complete AHR handrubbing achievement was 2.4 (±0.5). By protocol, the UV cabinet was not proposed to the control group on the first year, and their first rate of complete AHR handrubbing under UV light was only available during their second-year session. This rate was not significantly different from the first year intervention group assessment (before supervised personal feedback) (*n* = 31/102, 30.4% versus *n* = 43/140, 30.7%, *p* = 0.96).

### Influence of supervised personal feedback on the level of completeness with the WHO’s handrubbing technique

After didactic theoretical training and visual demonstrations, the level of completeness for AHR handrubbing according with the WHO’s HH procedure (AHR handrubbing technique) was 63.8% during the first year and 56.7% for the second year (*p* = 0.17). The groups’ rates of compliant handrubbing of the WHO’s HH procedure were similar between each compared year (Table 3).

**Table 3:** Visual assessment of the World Health Organization’s hand hygiene procedure on first year (baseline before training) and on second year (after training)

	Variable	Control group *n* = 102	Intervention group (Supervised personal feedback) *n* = 140	*P*-value
**YEAR 1 BASELINE BEFORE TRAINING**	Fully respected procedure (%)	58.9	66.9	0.22
	Palm to palm application (%)	97.8	98.6	0.66
	Right palm over left dorsum with interlaced fingers application + Palm to palm with fingers interlaced application (%)	92.2	95.0	0.40
	Backs of fingers to opposing palms with fingers interlocked (%)	85.6	89.9	0.32
	Rotational rubbing of left thumb clasped in right palm and vice versa (%)	86.7	91.4	0.26
	Rotational rubbing, backwards and forwards with clasped fingers of right hand in left palm and vice versa (%)	85.6	87.1	0.75
	Duration ≥20 s (%)	86.7	92.1	0.18
**YEAR 2 AFTER TRAINING**	Fully respected procedure (%)	50.5	61.3	0.09
	Palm to palm application (%)	98.0	97.1	0.66
	Right palm over left dorsum with interlaced fingers application + Palm to palm with fingers interlaced application (%)	95.0	90.5	0.19
	Backs of fingers to opposing palms with fingers interlocked (%)	71.3	80.3	0.11
	Rotational rubbing of left thumb clasped in right palm and vice versa (%)	79.2	82.5	0.52
	Rotational rubbing, backwards and forwards with clasped fingers of right hand in left palm and vice versa (%)	90.1	91.2	0.76
	Duration ≥20 s (%)	92.1	96.3	0.16

*p*-value: chi-2 Pearson test

### Influence of the UV auto-training on the handrubbing compliant with the WHO’s HH opportunities

During the second year, the rate of full compliance with the WHO’s HH opportunities (“the five moments for HH in health care”) in the intervention group was 58.1% versus 42.4% in the control group (*p* = 0.018) (Table 4). Despite the lack of statistical significance, moment 1 of “WHO’s five moments for HH in health care” (Before touching the patient), moment 3 (after the gloves) and moment 4 (after touching the patient) tend to be higher in the intervention group.

**Table 4:** Respect of the WHO’s hand hygiene opportunities on second year (“the five moments for HH in health care”)

Variable	Control group *n* = 102	Intervention group (Supervised personal feedback) *n* = 140	*p*-value
Full compliance (all the HH opportunities performed) (%)	42.4	58.1	0.018
- Entry/before preparing the material (respect of moment 2) (%)	100	100	–
- Before touching the patient (respect of moment 1) (%)	77.8	85.3	0.14
- Before glove for puncture (respect of moment 2) (%)	80.9	79.8	0.84
- After gloves for puncture (respect of moment 3)	74.7	80.9	0.26
- After gloves for material evacuation (respect of moment 3) (%)	76.8	81.6	0.36
- After touching the patient before exit (respect of moment 4) (%)	75.8	84.6	0.09

*p-value: chi-2 Pearson test*

The moments refer to the WHO’s indications for hand hygiene (“the five moments for HH in health care”)

Compliance at the entry is 100% in both groups because assessment of application of the AHR under UV light was done at this step

Each student was evaluated during the scenario-based learning for an arterial puncture (first attempt). On a standardized document, the tutor visually assessed and recorded their respect of the WHO’s hand hygiene opportunities during the simulation session

Full observance corresponded that the student performed all (6) the HH opportunities

*Abbreviations: HH hand hygiene, UV Ultraviolet, AHR alcohol-based handrub*

In the logistic regression, the intervention group was the only variable associated with complete compliance of the WHO’s HH opportunities (Table 5). The HR for the intervention group was 1.88 (95% CI: 1.10–3.21, *p* = 0.02). Even after forcing the variable “women” in the model, the intervention group’s full observance was significantly associated with the WHO’s HH objectives (data not shown).

**Table 5:** Factors associated with the respect of the WHO’s hand hygiene opportunities (“the five moments for HH in health care”) on second year

Variable	Incomplete respect (*n* = 117)	Full respect (*n* = 125)	*p*-value	Hazard ratio	95% confidence interval	*p*’-value
	Univariate			Logistic regression		
Personal feedback with UV cabinet (%)	50.0	65.3	0.016	1.88	1.10–3.21	0.02
Female gender (%)	60.5	67.8	0.247	–	–	–
Age ± SD (years)	21 ± 2.6	21 ± 1.8	0.254	–	–	–
New Hand hygiene formation (%)	41.6	33.1	0.179	0.70	0.40–3.20	0.20
Extra-university use of UV cabinet (%)	22.1	21.2	0.863	–	–	–
Infectious disease unit traineeship (%)	17.7	17.8	0.985	–	–	–
Surgical unit traineeship (%)	66.1	58.5	0.235	–	–	–
Intensive care unit traineeship (%)	11.5	12.7	0.779	–	–	–
Care of a patient needing contact precautions (%)	71.7	72.9	0.839	–	–	–
Care of a patient hospitalized for nosocomial infection (%)	17.7	13.7	0.401	–	–	–
Familiarity with alcoholic solution for HH (%)	73.5	82.2	0.109	0.55	0.88–3.18	0.11

*p value =* comparison by univariate test between the group with incomplete respect versus the group with complete respect of the WHO’s HH opportunities

*p’-value: p-value* by the multivariate logistic regression

*Abbreviations: SD standard deviation, UV Ultraviolet, HH Hand hygiene*

## Discussion

With the participation of second and third year undergraduate medical students, we assessed if the HH was enhanced by the addition of supervised personal feedback with a fluorescent AHR and UV light inspection system (UV cabinet) during simulation scenario-based for training. Our results showed that when HH educational program include the use of a supervised personal feedback with a fluorescent AHR and UV light inspection system, the completeness with the AHR handrubbing and the compliance with WHO’s HH opportunities were sustainably improved. We hypothesize that the use of fluorescent-AHR and UV-cabinet allows a direct personal feedback for the student. This feedback could have an important impact on the memorization process for the handrubbing technique allowing the complete application of the AHR. With the UV-cabinet, there would be a playful aspect which could facilitate the learning and memorization process. Further studies are needed to investigate this hypothesis.

Kaur *et al* underlined a lack of a rigorous evaluation of tools and educational material for medical students [8]. In our study, the presence of a control group allowed individualization of the proper effect of the intervention group inside our multimodal educational program.

Despite this multimodal program, overall, there was a low rate of completeness of AHR handrubbing (60% in the intervention group and 30.4% in the control group). Our hypothesis was that long-term outcome evaluations revealed a progressive decline in compliant handrubbing and regularly supervised personal feedback would be needed to receive the procedure’s benefits.

Some studies previously reported using supervised personal feedback with UV light inspection systems in the medical students’ training programs [11, 12, 16]. In all these studies, the absence of a control group did not allow individualizing the proper effect of feedback with UV within the rest of the training program. Scheithauer et al. observed that third-year students receiving timely training of HH had an immediate (22%) reduction in incomplete handrubbing after monitoring with the UV cabinet [11]. The baseline rate of completeness of AHR handrubbing after the first UV training was 29%. This was similar to the baseline rate observed in the first-year intervention group (30.7%), with ongoing training yielding a positive impact on outcomes. Lehotsky et al. observed that third-year students in the basic surgical techniques class used a UV based system for assessment and auto feedback immediately after receiving HH education [16]. This study reported a completeness rate of handrubbing of 61.8%, which is also very similar to the rate observed during the second-year intervention group (60%). Vanyolos et al. observed 285 medical students who were included in an educational program on “basic surgical techniques” which included a lecture on HH and a training with groups of 5 to 7 students [12]. The completeness rate of handrubbing wasn’t recorded immediately after the first application. The authors reported a rate of complete AHR application of 51.4% at week 14 and 74.3% at week 10 after the intervention [12].

Unexpectedly, when the tutor visually assessed the quality of the WHO’s handrubbing procedure without UV cabinet, the difference in the completeness rate of handrubbing in the second-year students between the two groups was not significant (Tables 3, 61.3% versus 50.5%, *p* = 0.096; intervention vs. control groups, respectively). This may be due to a lack of sensibility of single visual assessment without UV light to detect the forgotten steps of the WHO’s HH procedure. It may also be explained by integrating the missed skin areas despite correct procedures and consequently, the addition of supplementary movements to reach the completeness of handrubbing. The WHO’s HH procedure was designed to ensure homogenous hand surface coverage by applied AHR. However, it is not user friendly and recent studies question its adaptability. Indeed, when monitored, HCW compliance with all six steps of the procedure, is low with the last steps (fingertips and thumbs) being the most frequently missed [16–18]. Alternative methods with equivalent bacterial effectiveness have been proposed [19, 20]. However, more research is needed in order to validate this conclusion.

At the beginning of the second simulation session, there was a non-significant trend towards a decrease in the rate of complete compliance of the HH procedure in both groups when no technique review had been proposed. This suggests that, as with other HCWs, regular training is obligatory to comply with HH practices [21].

Our results suggested that the intervention group had a positive influence on compliant handrubbing with the WHO’s HH opportunities during the simulation scenario-based learning. This was in accordance with the Higgins et al study, which reported a sustained improvement of HH compliant handrubbing from 20 to 58% within a year after implementation of a personal feedback tool using gaming technology with an automated auditing and training unit [22]. This was however not confirmed in the Kwok et al. study [23]. This effect may be related to the role of personal feedback in awareness of HH’s importance to prevent cross transmission.

Our study has several limitations. Firstly, since we wanted to ensure that control clusters wouldn’t benefit from any feedback with the UV cabinet, we didn’t assess the completeness of their WHO’s HH procedure with UV cabinets during the first year. Consequently, their first completeness rate for the handrubbing under a UV light was only available during their second year. This rate was not significantly different from the intervention group’s rate (before supervised personal feedback) evaluated first year (data not shown). Secondly, our UV assessment cabinet didn’t provide photo storage after fluorescent AHR use for a retrospective, exhaustive analysis of the missed locations with a planimetry system. Thus, we chose to divide the AHR application as complete or not, since we could not study the relationship between the steps forgotten during the HH procedure and the areas missed. This information could have helped us better precisely understand the change in the AHR techniques induced by the UV lamp feedback. Thirdly, the personal experiences of students between the 1st and the 2nd year impacted the study outcome and represent confounding factors. On the 2nd year, we asked the students about their personal feedback for these confounding factors. However, despite our methodological cautions and the multivariate analysis, we cannot exclude that the students forgot to specify some confounding factors (memory bias). Moreover, our study did not allow to know if the students who performed AHR complete application more often remember extra-university use of UV cabinet, or, if the students using the extra-university of UV cabinet performed AHR complete application better. Finally, whether or not our results can be extrapolated in healthcare settings is speculative because students have a higher exposure to negative role models, as a poor compliance of the WHO’s HH recommendations by some heath care workers.

## Conclusion

A key component of the undergraduate medical students’ training for hand hygiene should include supervised personal feedback with UV cabinets in handrubbing procedures to improve theirs compliance rate with WHO’s recommendations (handrubbing procedure and hand hygiene opportunities).

## Supplementary information

**Additional file 1.** World health organization’s hand hygiene procedure with Alcohol-based handrub.

**Additional file 2.** Second year questionnaire about hand hygiene cursus in the interval between the two facilitation sessions.

**Additional file 3.** Standardized assessment form for Alcohol-based handrub application.

**Additional file 4.** Standardized assessment form for Alcohol-based handrub opportunities.

## Data Availability

The datasets used and analyzed during the current study are available from the corresponding author on reasonable request.

## Abbreviations

AHR: Alcohol-based handrubs
CI: Confidence interval
HH: Hand hygiene
IQR: Interquartile range
UV: Utraviolet
UVc: Ultraviolet cabinet
WHO: World Health Organization
