# Supplementary material for: Using an ultraviolet cabinet improves compliance with the World Health Organization’s hand hygiene recommendations by undergraduate medical students: a randomized controlled trial
Source: Antimicrob Resist Infect Control. 2020 Sep 3;9:147. doi: 10.1186/s13756-020-00808-4 (PMC7469265; doi:10.1186/s13756-020-00808-4)
Supplement: Supplementary file 2 — Additional file 2. Second year questionnaire about hand hygiene cursus in the interval between the two facilitation sessions. [file 13756_2020_808_MOESM2_ESM.docx]

**Additional file 2**

Second year questionnaire about hand hygiene cursus in the interval between the two facilitation sessions.

Concerning your Hand Hygiene practice in the interval between your first and second facilitation session, please complete this quick questionnaire:

|  | Yes | No |
| --- | --- | --- |
| Did you have any new Hand hygiene formation? |  |  |
| Did you experiment extra-university use of ultraviolet cabinet? |  |  |
| Did you have a traineeship in Infectious disease unit? |  |  |
| Did you have a traineeship in surgical unit? |  |  |
| Did you have a traineeship in intensive care unit? |  |  |
| Did you have the Care of at least one patient needing contact precautions? |  |  |
| Did you have the Care of a patient hospitalized for nosocomial infection? |  |  |
| Did you mainly use alcoholic solution for hand hygiene? |  |  |
