# Supplementary figures and images for: Using an ultraviolet cabinet improves compliance with the World Health Organization’s hand hygiene recommendations by undergraduate medical students: a randomized controlled trial
Source: Antimicrob Resist Infect Control. 2020 Sep 3;9:147. doi: 10.1186/s13756-020-00808-4 (PMC7469265; doi:10.1186/s13756-020-00808-4)

**Additional file 4**

Standardized assessment form for Alcohol-based handrub opportunities.


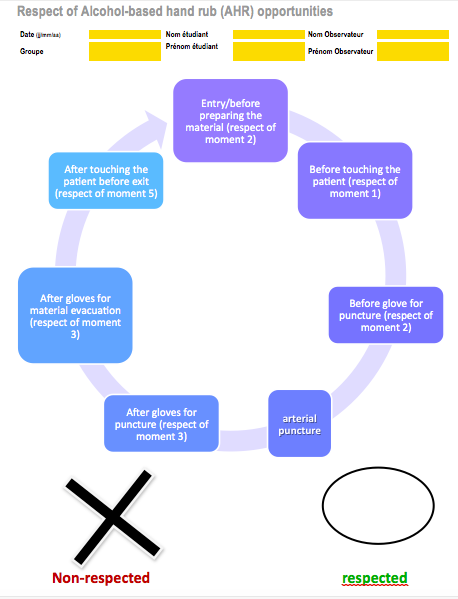

Supplement: Supplementary file 4 — Additional file 4. Standardized assessment form for Alcohol-based handrub opportunities. [file 13756_2020_808_MOESM4_ESM.docx]
